# Estudio de estabilidad de los controles de calidad internos almacenados en el módulo de refrigeración automatizado de Atellica Solution

**DOI:** 10.1515/almed-2022-0074

**Published:** 2023-02-20

**Authors:** Patricia Rayo Hidalga, Carlos Domingo Bautista, Rosa Fernández Bonifacio, José Luis Bedini, Naira Rico

**Affiliations:** Área de Preanalítica, Centro Diagnóstico Biomédico, Hospital Clínic, Barcelona, España; Laboratorio Core, Centro Diagnóstico Biomédico, Hospital Clínic, Barcelona, España

**Keywords:** Atellica Solution, control de calidad, estabilidad, límite de cambio total, módulo de refrigeración

## Abstract

**Objetivos:**

Atellica Solution (AS) es una plataforma que permite incorporar módulos de bioquímica e inmunoanálisis y almacenar controles de calidad internos (CC) en su interior gracias al módulo de refrigeración (MR). El objetivo del estudio es analizar el tiempo máximo de estabilidad de los CC almacenados en su interior.

**Métodos:**

Se analizaron 48 magnitudes mediante materiales CC: Liquid Assayed Multiqual (MQ), Liquichek Immunology (LI), Liquichek Lipids (LL), y Liquichek Urine Chemistry (UC). La variación en los resultados (Xt %) se calculó comparando el promedio del análisis realizado en cada momento (Xt) con el promedio realizado en el momento inicial del estudio (Xo), y se expresó como un cambio porcentual: (Xt%) = (Xt/Xo) × 100. La estabilidad se evaluó de acuerdo con el límite de cambio total (LCT) el cual combina la variabilidad analítica y biológica: LCT = ±√((1,65 × CVa)^2^ + (0,5 × CVb)^2^)

**Resultados:**

Un total de 40 de las 48 magnitudes estudiadas fueron estables al finalizar el estudio. En el caso del CC MQ y el UC 32 de las 39 magnitudes fueron estables durante los 15 días del estudio, excepto fosfatasa alcalina, aspartato aminotransferasa, calcio, lactato deshidrogenasa y bilirrubina total en MQ y cloro y glucosa en UC. En los CC LI, LL 8 de las 9 magnitudes fueron estables durante los 20 días del estudio, a excepción de la transferrina en LI.

**Conclusiones:**

El módulo de refrigeración de Atellica Solution es un sistema fiable para mantener almacenados los materiales de control de calidad.

## Introducción

Los laboratorios clínicos tienen una gran presión asistencial ya que deben analizar un elevado número de muestras en un tiempo adecuado para que los clínicos puedan realizar el diagnóstico y seguimiento de los pacientes. Además, la calidad de los resultados debe estar asegurada mediante la utilización de controles internos y externos.

Atellica Solution es una plataforma de Siemens Healthineers (Tarrytown, NY) que permite combinar módulos de bioquímica e inmunoanálisis. Una de las ventajas de este analizador es la capacidad de almacenar controles de calidad internos (CC) y calibradores en su interior, gracias al módulo de refrigeración (MR) que se sitúa dentro del *sample handler* o módulo de gestión de muestras, lo cual permite automatizar el procesamiento de los CC. El MR mantiene la temperatura entre 2–8 °C y permite almacenar un máximo de 60 CC y/o calibradores. Los contenedores utilizados son tubos de 5 mL que se cargan destaponados, el MR dispone de unas tapas magnéticas que cubren, de forma individual, la posición donde se coloca el tubo evitando la evaporación. Los CC pueden almacenarse durante varios días, el tiempo es definido por el usuario.

La estabilidad de los materiales CC debe estar asegurada durante el tiempo que se utilicen, ya que la pérdida de estabilidad puede conllevar una mayor repetición y verificación de los CC, causando un trabajo innecesario, enlenteciendo el procesamiento de las muestras y produciendo un mayor gasto de controles y reactivos. El proveedor de los CC suele establecer unas recomendaciones sobre el tiempo máximo de estabilidad en el módulo de refrigeración, no obstante siempre es recomendable que cada laboratorio haga su propia evaluación. Además, la norma ISO 15189:2012 recomienda que se analicen periódicamente los materiales de control interno según la estabilidad del sistema [1]. El objetivo del estudio es analizar, para cada magnitud bioquímica, el tiempo máximo de estabilidad de los CC almacenados en el interior del MR de AS.

## Materiales y métodos

### Selección y procesamiento de muestras

El estudio se realizó en el Laboratorio Core del Hospital Clínic de Barcelona en Febrero de 2019. Durante el estudio se utilizó siempre el mismo analizador AS, se mantuvieron los mismos lotes de reactivo y se intentó minimizar el número de calibraciones priorizando la calibración de lote a la calibración de cartucho siempre que fuera posible.

Se utilizaron cuatro materiales de CC de dos niveles cada uno de ellos: Liquid Assayed Multiqual (MQ) niveles 2 y 3 del lote 45800, Liquichek Immunology (LI) niveles 1 y 2 del lote 66370T, Liquichek Lipids (LL) niveles 1 y 2 de los lotes 57560 y 57550 respectivamente, y Liquichek Urine Chemistry (UC) niveles 1 y 2 del lote 66790T; todos ellos del proveedor Bio-Rad Laboratories (Irvine, CA). Durante todo el estudio se mantuvieron los mismos lotes de CC. Los viales de CC MQ y LL fueron traspasados a tubos de 5 mL donde se dispensaron 3 mL de material en cada uno de ellos. En el caso de los CC LI y UC venían preparados en formato tubo con un volumen de 3 mL y 4 mL respectivamente. Para realizar el estudio, se guardaron en el MR 5 tubos de cada nivel del CC MQ y 3 tubos de cada nivel de los CC UC, LI y LL, el número de tubos se estableció en base al panel de pruebas a solicitar, calculando un mayor consumo del CC MQ. Los CC permanecieron almacenados en el MR durante 15 días en el caso del MQ y UC, y 20 días el LI y LL. La diferencia en el tiempo de almacenamiento se debió al consumo del material control, en MQ y UC se analizó un panel de magnitudes más amplio que LI y LL.

Cada CC se analizó por triplicado una vez al día para evitar un posible error aleatorio de pipeteo y obtener resultados fiables. Antes de cada serie analítica, se analizaron CC para asegurar la comparabilidad de los resultados. Aquellas magnitudes que no cumplían con los criterios de calidad establecidos en nuestro laboratorio (media ±2 × desviación estándar), fueron calibrados y nuevamente controlados. El tiempo cero se consideró el momento en que se cargaron por primera vez los CC, el valor obtenido en ese momento se consideró el valor de referencia.

Para valorar si la imprecisión analítica de los métodos utilizados en AS era adecuada, se calculó el CVa, para cada magnitud, a partir de los materiales CC analizados para asegurar la comparabilidad de los resultados durante el estudio. Como criterio de aceptación se utilizaron las especificaciones de calidad basadas en la variabilidad biológica tal como recomienda el documento elaborado por la Comisión de Calidad Analítica de la SEQC^ML^ [2].

### Evaluación de la estabilidad

Se estudiaron 48 magnitudes bioquímicas. En el control MQ: ácido úrico (UA), alanina aminotransferasa (ALT), albúmina (ALB), amilasa (Amylas), aspartato aminotransferasa (AST), bilirrubina directa (DBil), bilirrubina total (TBil), calcio (Ca), cloro (CL), colesterol total (TChol), colesterol-HDL (cHDL), colesterol-LDL (cLDL), creatinina (Crea), creatina cinasa (CK), fosfatasa alcalina (ALP), fosfato (IP), glucosa (Glu), gamma-glutamiltransferasa (GGT), hierro (Iron), lactato deshidrogenasa (LDH), magnesio (Mg), potasio (K), proteínas totales (TP), sodio (Na), triglicéridos (Trig) y urea nitrogenada (UN). En el control LI: haptoglobina (Hapt), transferrina (Trf), proteína C reactiva (CRP), prealbúmina (preALB), complemento C3 (C3) y complemento C4 (C4). En el control LL: apolipoproteína A1 (APO A1), apolipoproteína B (APO B) y proteína C reactiva de alta sensibilidad (hsCRP). Por último, en el control UC: microalbúmina (microALB), Amylas, Ca, CL, Crea, Glu, IP, K, Mg, Na, UA, UN y TP.

Después de cada una de las series analíticas, las alícuotas de CC fueron automáticamente tapadas y almacenadas de nuevo en el MR de AS.

### Análisis de los datos

El porcentaje de variación en los resultados (Xt %) se calculó comparando el promedio del análisis por triplicado realizado en cada momento “t” (Xt) con el promedio del triplicado realizado en el momento inicial del estudio (Xo) y se expresó como un cambio porcentual: Xt % = (Xt/Xo) × 100 [3]. Como criterio para definir los límites de estabilidad de cada magnitud se utilizó el límite de cambio total (LCT), el cual combina la variabilidad analítica y biológica: LCT = ±√((1,65 × CVa)^2^ + (0,5 × CVb)^2^). El criterio de LCT ha sido utilizado en estudios previos de estabilidad realizados por nuestro grupo de trabajo [3, 4]. El CVa es el coeficiente de variación analítico para cada magnitud y se calculó utilizando los datos de CC internos obtenidos en los 5 meses previos a la fecha del estudio y el CVb es la variabilidad biológica intraindividual para cada magnitud obtenida de la base de datos de la *European Federation of Clinical Chemistry and Laboratory Medicine* (EFLM) [5]. En el caso de las magnitudes no publicadas en la EFLM, los CVb se obtuvieron de las especificaciones deseables de la base de datos de variación biológica de la Dra. Carmen Ricos [6] (Tabla 1). Para las magnitudes CL y Glu del control UC en el cálculo del LCT utilizamos únicamente el criterio de variabilidad analítica (CVa) ya que no se disponía de CVb de CL y Glu en orina en ninguna base de datos. En este último caso la fórmula aplicada fue: LCT = ±√((1,65 × CVa)^2^).

**Tabla 1:** Coeficientes de variación analítica y biológica de las magnitudes estudiadas.

Magnitud	CVa% (s) 5 meses	Cva% (o) 5 meses	CVb% (s)	CVb% (o)
ALB	1,9	8,4	2,6	
ALT	1,5		10,1	
ALP	2,3		5,3	
Amylas	1,1	2,1	6,6	94^a^
APO A1	2,9		5,4	
APO B	4,2		7,4	
AST	2,3		9,6	
C3	2,9		4,6	
C4	1,7		6,9	
Ca	2,3	3	2,1^a^	27,5^a^
TChol	1,3		5,3	
CK	1,8		15	
CL	1,3	1,4	1,1	–
Crea	2,2	1,5	4,5	24
cHDL	3,1		5,8	
DBil	2,8		36,8^a^	
cLDL	1,6		8,3	
GGT	2,7		9,1	
Glu	0,9	1,6	5	–
Hapt	2,3		8,6	
hsCRP	11,9		58,9	
IP	1,6	2,1	7,8	18^a^
Iron	1		26,5^a^	
K	0,7	1,8	4,1	24,4^a^
LDH	2,2		5,2	
Mg	3,3	5	2,9	38,3^a^
microALB		7,9		36
Na	0,5	1,8	0,5	28,7^a^
Tbil	1,7		21,8^a^	
CRP	3,7		34,1	
preALB	5,5		10,9^a^	36^a^
TP	2,1	2	2,6	35,5^a^
Trig	1,6		20	
Trf	2,8		3,9	
UA	0,8	2,3	8,6^a^	16,8^a^
UN	1,7	3,4	13,9	17,4^a^

CVa% (s), coeficiente de variación analítica en suero; CVa% (o), coeficiente de variación analítica en orina; CVb% (s), variabilidad biológica intraindividual en suero; CVb% (o), variabilidad biológica intraindividual en orina; ^a^valores obtenidos de la base de datos de variación biológica de la Dra. C. Ricos; ALB, albúmina; ALT, alanina aminotransferasa; ALP, fosfatasa alcalina; Amylas, amilasa; APO A1, apolipoproteína A1; APO B, apolipoproteína B; AST, aspartato aminotransferasa; C3, complemento C3; C4, complemento C4; Ca, calcio; TChol, colesterol total; CK, creatina cinasa; CL, cloro; Crea, creatinina; cHDL, colesterol-HDL; DBil, bilirrubina directa; cLDL, colesterol-LDL; GGT, gamma-glutamiltransferasa; Glu, glucosa; Hapt, haptoglobina; hsCRP, proteína C reactiva de alta sensibilidad; IP, fosfato; Iron, hierro; K, potasio; LDH, lactato deshidrogenasa; Mg, magnesio; microALB, micro albúmina; Na, sodio; TBil, bilirrubina total; CRP, proteína C reactiva; preALB, prealbúmina; TP, proteínas totales; Trig, triglicéridos; Trf, transferrina; UA, ácido úrico; UN, urea nitrogenada.

Se consideró pérdida de estabilidad cuando el Xt % excedía el LCT. Se seleccionó como tiempo máximo de estabilidad para una magnitud en concreto aquel momento a partir del cual el Xt %, para uno de los niveles o bien para ambos, se excedía del LCT de forma consecutiva durante más de 2 días. A partir de ese momento el CC no era estable para esa magnitud en concreto.

## Resultados

La imprecisión analítica (CVa) que mostraron los materiales CC analizados durante los 15 días del estudio en el caso del MQ y UC, y los 20 días del estudio en el caso del LI y LL, así como el CVa de los 5 meses previos, fueron aceptables. El criterio de aceptación se basó en el cumplimiento de las especificaciones de calidad basadas en variabilidad biológica (VB). Todas las magnitudes cumplieron con las especificaciones óptimas excepto ALP, Crea, LDH y preAlb que cumplieron con las especificaciones deseables; ALB, APO A1, APO B, C3, cHDL y Trf que cumplieron las especificaciones mínimas y Ca, CL, Mg y Na que no cumplían especificaciones basadas en VB pero sí que presentaban un CVa inferior al de su grupo par por método. Este último dato fue obtenido del programa de calidad externo de la FPCQLC del año 2019.

**Figura 1:**
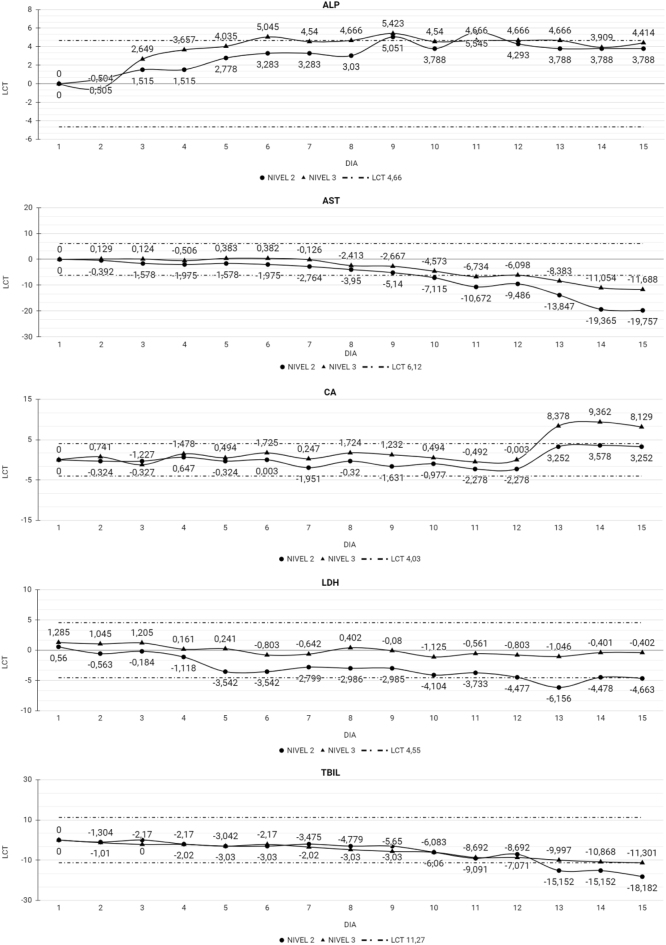
Magnitudes que perdieron la estabilidad en el CC MQ. Magnitudes que perdieron la estabilidad en el CC MQ tras los 15 días de estudio. ALP, fosfatasa alcalina; AST, aspartato aminotransferasa; CA, calcio; LDH, lactato deshidrogenasa; TBIL, bilirrubina total; LCT, límite de cambio total; Nivel 2, nivel medio del CC MQ; Nivel 3, nivel alto del CC MQ.

**Figura 2:**
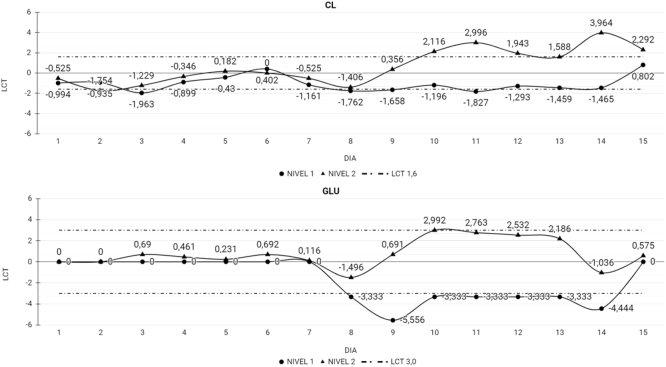
Magnitudes que perdieron la estabilidad en el CC UC. Magnitudes que perdieron la estabilidad en el CC UC durante los 15 días del estudio. CL, cloro; GLU, glucosa; LCT, límite de cambio total; Nivel 1, nivel bajo del CC UC; Nivel 2, nivel alto del CC UC.

Las Tablas 2 y 3 muestran el Xt % para cada magnitud por día además del LCT, indicando en qué momento el Xt % excedió el LCT, resultando en una pérdida de estabilidad de la magnitud. Un total de 40 de las 48 magnitudes estudiadas fueron estables al finalizar el estudio. En el caso de los CC MQ y el UC, 32 de las 39 magnitudes fueron estables durante los 15 días del estudio. En el CC MQ ALP, AST, Ca, LDH y TBil perdieron la estabilidad el día 8 (ALP), 10 (AST) y 13 (Ca, LDH y TBil). En la Figura 1 se muestra el comportamiento de las magnitudes con el paso del tiempo, ALP y Ca presentan una tendencia en ascenso, mientras que AST, LDH y TBil la tendencia es en descenso. En el caso del CC UC CL y Glu perdieron la estabilidad el día 8 con un comportamiento sin una tendencia clara (Figura 2).

**Tabla 2:** Controles MQ y UC, cambio porcentual para cada magnitud durante los días del estudio y valor de LCT.

CC MQ	D 1	D 2	D 3	D 4	D 5	D 6	D 7	D 8	D 9	D 10	D 11	D 12	D 13	D 14	D 15	LCT
ALB LOW	0	−0,92	0	−0,93	0	−2,78	−1,85	−0,93	−0,93	−0,93	0	0	−1,85	−1,85	0,926	3,43
ALB HIGH	−1,48	0,74	0	0	0	0	0	0	0	0	0	0	0	−0,74	0	3,43
ALP LOW	0	0,505	1,515	1,515	2,778	3,283	3,283	3,03	5,051	3,788	5,545	4,293	3,788	3,788	3,788	4,66
ALP HIGH	0	−0,50	2,649	3,657	4,035	5,045	4,54	4,666	5,423	4,54	4,666	4,666	4,666	3,909	4,414	4,66
ALT LOW	1,118	1,616	3,715	0,808	−3,13	1,207	−2,32	0,853	0,519	−1,31	−2	−0,89	−5,48	−4,06	−4,42	5,64
ALT HIGH	−0,48	−1,13	−1,45	−2,42	−1,45	−2,26	−2,42	−3,06	−2,90	−2,90	−4,35	−2,90	−6,46	−3,71	−5,00	5,64
Amylas LOW	0,002	0,509	0,256	0,002	−0,25	–	−0,51	0,002	−0,25	−0,25	−0,51	0,254	−1,27	0,003	0,254	3,83
Amylas HIGH	0,72	1,082	0,841	0,36	0,602	–	−0,72	0,48	0,481	0,481	0,121	0,962	−0,48	−0,60	0,602	3,83
AST LOW	0	−0,39	−1,58	−1,97	−1,58	−1,98	−2,76	−3,95	−5,14	−7,11	−10,7	−9,49	−13,8	−19,4	19,76	6,12
AST HIGH	0	0,129	0,124	−0,51	0,383	0,382	−0,13	−2,41	−2,67	−4,57	−6,73	−6,10	−8,38	−11,0	−11,7	6,12
Ca LOW	0	−0,324	−0,327	0,647	−0,324	0,003	−1,951	−0,32	−1,631	−0,977	−2,278	−2,278	3,252	3,578	3,252	4,03
Ca HIGH	0	0,741	−1,23	1,478	0,494	1,725	0,247	1,724	1,232	0,494	−0,49	−0,00	8,378	9,362	8,129	4,03
TChol LOW	0	−0,17	0,355	0,355	0,176	1,417	1,77	1,063	1,418	1,062	0,531	1,417	−2,12	−1,42	−0,53	3,41
TChol HIGH	0	0,122	0,363	0,363	0,726	2,054	2,054	1,087	1,088	1,571	0,967	1,571	−1,69	−0,60	−0,36	3,41
CK LOW	0,619	0,62	−0,25	−0,49	0,124	−1,11	−1,85	−1,47	−0,86	−1,48	−2,59	−1,48	−3,82	−2,34	−1,85	8,1
CK HIGH	−1,13	−0,15	−0,71	−1,23	−0,92	−1,54	−2,00	−2,46	−2,67	−2,47	−2,98	−2,62	−4,11	−3,49	−2,93	8,1
CL LOW	−0,33	−0,03	−0,2	−1,40	−0,53	−0,4	−0,07	−0,13	0,067	−1,13	−0,3	−0,3	−1,00	−0,4	−0,03	2,27
CL HIGH	1,137	0,287	0,002	0	−0,28	−0,28	−0,28	−0,28	−0,56	0,002	−0,56	−0,57	−0,85	−1,13	−1,13	2,27
Crea LOW	0,172	−0,17	−1,03	−0,86	0,172	–	−1,20	−0,86	−1,72	−1,55	0,696	−0,68	−3,1	−1,20	0,005	4,4
Crea HIGH	0,261	0,52	0,983	0,056	–	–	−0,76	−0,40	−0,61	0,312	−0,30	−0,20	−1,39	−1,29	−0,15	4,4
cHDL LOW	0	−2,70	−2,70	−2,70	−0,90	0	0	−2,70	−1,80	−3,60	−5,40	−5,40	−5,40	−5,40	−5,40	5,89
cHDL HIGH	0	−1,74	−2,32	−3,49	−0,57	0,585	0,01	−2,32	−2,89	−5,81	−5,81	−5,81	−3,48	−4,64	−4,06	5,89
DBIL LOW	2,381	7,143	7,143	7,143	7,143	7,143	7,143	7,143	0	−2,38	−2,38	−7,14	−7,14	−7,14	−9,52	19,01
DBIL HIGH	0	3,846	3,846	3,846	6,41	5,128	1,282	3,846	0	−1,282	0	−1,282	−3,846	−3,846	−3,846	19,01
cLDL LOW	0,273	0,817	−0,54	0	−0,27	0,815	0,546	0,273	−0,27	0,544	0,549	0,817	0,544	0,546	−0,27	4,98
cLDL HIGH	2,536	2,929	1,561	1,367	0,002	0,197	0,978	0,585	0,587	0,976	1,369	1,757	2,345	0,975	1,362	4,98
GGT LOW	3,35	2,142	4,194	3,82	2,136	3,408	2,564	2,147	1,72	3,397	3,381	3,82	2,116	0,87	2,537	6,39
GGT HIGH	0,769	0,509	1,793	1,308	1,811	1,799	2,32	0,521	1,29	1,29	2,308	2,326	−0,006	3,083	2,829	6,39
Glu LOW	0	0	1,488	1,488	1,786	2,083	1,786	0,893	1,19	1,19	1,786	1,786	−1,786	−0,893	−1,19	3,09
Glu HIGH	0	−0,199	1,397	1,994	2,293	2,892	2,592	1,794	1,695	1,595	1,795	2,093	−1,695	−1,196	−0,797	3,09
P LOW	0	0,813	0,813	−0,813	0,813	1,626	0	0	0,813	0,813	1,626	2,439	2,439	2,439	0	4,72
P HIGH	−0,457	0,913	0,457	1,37	1,826	1,37	1,37	0,913	0,457	1,37	0,913	0,913	0,913	0,457	0,913	4,72
Iron LOW	0,218	0,218	0,437	0,003	0,436	0,437	0,219	1,525	1,089	1,303	2,391	0,652	−0,22	0,654	0,87	13,35
Iron HIGH	1,326	1,62	1,327	1,033	0,593	0,885	0,737	1,177	1,324	1,177	1,031	1,32	0,595	0,737	1,624	13,35
K LOW	0,44	0,177	0,438	0,613	0,525	0,264	−0,60	0,003	−0,34	0,267	−0,08	0,003	−0,52	0,612	0,959	2,37
K HIGH	0,228	0,773	0,183	0,545	0,864	0,546	0,091	0,273	−0,50	−0,23	0,272	0,091	−0,54	−0,14	0,228	2,37
LDH LOW	0,56	−0,56	−0,18	−1,12	−3,54	−3,54	−2,80	−2,99	−2,98	−4,10	−3,73	−4,48	−6,16	−4,48	−4,66	4,55
LDH HIGH	1,285	1,045	1,205	0,161	0,241	−0,80	−0,64	0,402	−0,08	−1,12	−0,56	−0,80	−1,05	−0,40	−0,40	4,55
Mg LOW	0	0	0	0	0	0	0	0	−4	0	1,333	0	−4	−1,33	0	5,71
Mg HIGH	−1,55	−0,74	0,038	−0,77	−1,55	−1,55	−2,36	0,038	−1,55	−0,74	−0,77	−1,55	−1,55	−0,74	−1,55	5,71
Na LOW	0,243	0,24	0,48	0,48	0,243	0,003	−0,47	−0,23	−0,47	0,247	0,007	−0,23	−0,47	0,48	0,713	0,92
Na HIGH	0,214	0,214	0,001	−0,64	0,214	−0,21	−0,42	0	−0,42	−0,42	−0,21	0,001	−0,64	0	0,214	0,92
TBil LOW	0	−1,01	0	−2,02	−3,03	−3,03	−2,02	−3,03	−3,03	−6,06	−9,09	−7,07	−15,1	−15,1	−18,2	11,27
TBil HIGH	0	−1,30	−2,17	−2,17	−3,04	−2,17	−3,47	−4,78	−5,65	−6,08	−8,69	−8,69	−9,99	−10,9	−11,3	11,27
TP LOW	0	−0,62	−0,62	0	0	0	−1,23	−1,23	−1,85	−1,23	−1,85	0	−0,62	0	1,235	3,73
TP HIGH	0,966	0	−0,48	0	0	0	−0,97	−0,48	−1,45	0	0	0	0,483	0,966	0,966	3,73
Trig LOW	0,939	0,941	1,413	1,648	2,118	2,59	2,589	2,354	3,06	4,94	4,237	3,766	4,237	5,411	5,412	10,37
Trig HIGH	1,225	1,684	2,725	2,75	3,055	3,212	2,75	2,598	3,981	3,362	3,666	3,667	3,513	3,975	5,04	10,37
UA LOW	−1,12	−0,56	−0,56	−1,11	−0,56	−0,56	−0,56	−0,56	−0,56	−0,56	−0,56	0,565	−0,56	1,13	1,13	4,55
UA HIGH	0,721	0,362	1,079	0,362	0,362	0,725	0	0,725	0,362	0,004	0,362	1,083	1,441	1,083	1,8	4,55
UN LOW	−0,79	−0,79	−2,4	−3,21	−3,21	−2,42	0	0,813	0,813	0,019	−0,79	0,019	−0,79	−0,79	−1,59	7,49
UN HIGH	0	−1,43	−2,86	−3,33	−3,33	−4,76	0,476	0	0	0	−1,43	0	−1,43	−2,86	−2,86	7,49

CC UC	Día 1	Día 2	Día 3	Día 4	Día 5	Día 6	Día 7	Día 8	Día 9	D-10	D-11	D-12	D-13	D-14	D-15	LCT
Ca LOW	−0,96	0	1,442	0	1,919	1,449	−1,43	0,483	−1,91	2,871	1,925	−0,48	2,374	−2,88	4,79	15,75
Ca HIGH	0,002	−0,28	0,572	0	1,714	0,855	−1,14	0,287	0,575	6,572	6,86	6,278	2,289	−2,57	2,574	15,75
CL LOW	−0,99	−0,93	−1,96	−0,90	−0,43	0,402	−1,16	−1,76	−1,66	−1,20	−1,83	−1,29	−1,46	−1,46	0,802	1,6
CL HIGH	−0,52	−1,75	−1,23	−0,35	0,182	0	−0,52	−1,41	0,356	2,116	2,996	1,943	1,588	3,964	2,292	1,6
Crea LOW	1,925	2,129	3,291	0,618	1,784	2,145	0,432	1,539	−1,01	−0,77	0,923	2,256	0,62	0,685	1,686	12,46
Crea HIGH	0,954	1,464	2,404	0,686	1,47	2,837	0,04	1,565	−0,02	2,267	4,065	5,399	4,372	-,272	2,004	12,46
Glu LOW	0	0	0	0	0	0	0	−3,33	−5,56	−3,33	−3,33	−3,33	−3,33	−4,44	0	3
Glu HIGH	0	0	0,69	0,461	0,231	0,692	0,116	−1,50	0,691	2,992	2,763	2,532	2,186	−1,04	0,575	3
IP LOW	0,749	0,739	3,591	4,439	4,817	4,851	4,32	1,733	0,638	0,749	1,737	4,197	2,607	2,842	3,454	9,47
IP HIGH	−0,24	−1,22	0,802	2,202	4,037	3,676	3,246	1,104	1,105	3,616	4,703	6,968	6,482	1,381	1,288	9,47
K LOW	1,021	−2,76	1,531	1,224	1,736	1,534	1,022	0,307	0,205	1,327	−1,53	0,92	0,918	−0,20	−0,41	12,25
K HIGH	1,735	1,734	1,831	1,66	3,1	1,213	2,646	2,105	4,553	7,046	9,004	7,649	7,638	7,186	3,149	12,25
Mg LOW	−1,41	−1,17	0,954	0,876	4,212	2,667	−11,5	−2,61	−1,26	−1,70	−4,98	−3,61	−3,46	−1,56	0,799	20,19
Mg HIGH	0,519	0,104	1,326	3,325	3,486	4,878	−9,31	0,226	1,972	4,063	4,065	4,904	3,972	−0,53	0,682	20,19
Na LOW	0,155	5,117	1,202	0,581	0,388	0,116	0,698	0,582	−0,35	0,97	4,11	0,931	0,776	0,505	1,435	14,4
Na HIGH	0,19	0,379	0,001	0,378	0,754	0,568	0,758	0,564	1,89	4,343	5,661	4,529	4,342	1,135	0,754	14,4
UA LOW	2,836	1,578	3,148	1,572	0,629	0,952	3,774	1,893	0,326	1,261	−0,31	0,003	−0,48	1,258	2,213	10,48
UA HIGH	1,852	1,092	2,156	0,777	1,081	2,326	1,7	2,156	1,7	4,156	3,692	4,152	4,002	1,852	1,386	10,48
microALB LOW	0	−1,08	−1,48	0,882	−10,7	−12,1	−10,5	–	−7,15	−1,37	−14,0	−12,8	−1,37	−6,85	4,107	22,23
microALB HIGH	0	0,004	−1,14	−0,38	−1,90	−2,29	−3,83	−2,67	−4,98	−1,90	−3,05	−4,58	−20,7	–	−20,7	22,23
UN LOW	2,668	2,322	5,753	2,293	1,576	4,603	−0,87	5,705	4,467	2,637	2,698	3,846	5,645	5,841	4,624	11,94
UN HIGH	3,303	2,077	5,391	1,496	1,534	3,311	0,797	5,962	6,762	7,219	8,869	6,978	7,357	1,99	3,941	11,94
PT LOW	−1,65	2,855	2,748	1,981	3,653	−1,65	2,714	−0,55	0,428	2,913	2,339	2,493	2,082	4,251	3,526	19,6
PT HIGH	−0,95	0,375	−0,74	−0,10	1,385	0,797	−0,21	0,267	1,647	3,396	3,079	3,604	2,334	−0,24	−1,37	19,6
Amylas LOW	−0,59	0,665	1,923	0,047	0,024	1,923	−0,62	0,665	−1,23	−2,52	−0,62	−1,23	−0,59	0,665	0,665	47,2
Amylas HIGH	0,594	0,446	0,741	0,15	0,889	0	0,296	1,187	1,927	3,852	4,594	5,483	4,89	0,445	1,187	47,2

D se refiere al día; LCT, límite de cambio total; “-“ no se dispone de resultado por un error del analizador.

**Tabla 3:** Controles LL y LI, cambio porcentual para cada magnitud durante los días del estudio y valor de LCT.

CC LL	Día 1	Día 2	Día 3	Día 4	Día 5	Día 6	Día 7	Día 8	Día 9	Día 10	Día 11	Día 12	Día 13	Día 14	Día 15	Día 16	Día 17	Día 18	Día 19	Día 20	LCT
APO-A LOW	1,8	0,6	0,6	2,1	1,8	1,5	1,5	0,9	1,3	0,6	0,3	0,9	2,5	0,0	1,8	3,7	3,7	3,7	2,5	4,9	**5.5**
APO-A HIGH	–	–	–	–	–	–	–	–	–	–	–	–	–	–	–	–	–	–	–	–	**5.5**
APO B LOW	1,9	−2,2	−1,5	−1,9	1,9	0,8	–	−3,4	4,1	6,7	6,0	3,7	4,5	4,0	4,1	3,7	1,1	1,9	2,2	4,1	**7,9**
APO B HIGH	−2,5	−1,0	−1,7	−1,0	1,1	−2,3	–	−2,8	3,9	2,6	2,6	3,7	2,9	3,3	3,5	6,1	4,6	−2,5	5,0	5,4	**7,9**
hsCRP LOW	0	−2,1	−4,2	8,3	12	6,2	6,2	6,2	10	8,3	8,3	10	12	25	8,3	4,1	6,2	6,2	10	8,3	**35**
hsCRP HIGH	−2,4	−1,2	−1,8	10,3	9,70	6,67	9,70	6,67	7,27	6,06	8,48	6,06	4,24	6,67	7,88	3,64	5,45	5,45	4,85	7,27	**35**

CC LI	Día 1	Día 2	Día 3	Día 4	Día 5	Día 6	Día 7	Día 8	Día 9	Día 10	Día 11	Día 12	Día 13	Día 14	Día 15	Día 16	Día 17	Día 18	Día 19	Día 20	LCT
Hapt LOW	0	−0,9	−1,4	−0,9	−0,5	0,5	0,5	−0,9	2,1	0,9	−0,5	−0,5	−0,5	0,9	0,9	−1,4	−3,8	−2,3	2,8	2,3	**5,8**
Hapt HIGH	0,2	−0,9	−1,1	−1,5	−0,9	−0,2	0,4	−1,8	4,0	3,8	3,1	2	3,8	2,0	3,6	2,9	0,2	−0,6	5,8	**6,2**	**5,8**
Trf LOW	−0,2	0	−0,9	0,4	0,4	1,1	1,5	−1,3	−0,6	−0,9	−0,4	−0,2	1,1	−2,6	−2,2	−1,7	−2,2	**−7,8**	**−7,8**	**−8,1**	**5,3**
Trf HIGH	0,8	1,4	1,4	0,0	1,1	2,7	2,9	0,3	0,3	0,4	0,1	0,8	1,9	−1,1	0,8	−0,4	−0,8	−5,2	**−5,9**	**−7,4**	**5,3**
CRP LOW	3,6	1,6	2,6	2,1	3,6	1,6	4,7	4,2	2,6	1,6	3,1	−0,5	2,3	0,5	0,5	1,6	0,5	1,6	2,6	4,7	**18**
CRP HIGH	2	3,3	2,6	1,1	2,5	3,7	3,6	2,7	1,9	0,9	1,9	0,3	2,7	1,4	−1,2	1,1	0,1	1,2	1,3	2,3	**18**
preALB LOW	−1,0	−0,8	−1,3	−2,9	−3,6	−4,2	2,6	−1,0	3,7	−1,3	−0,5	−1,0	1,3	1,6	−2,0	−1,6	1,3	0,5	−0,8	−2,8	**11**
preALB HIGH	−0,3	0,1	−0,6	0	−2	−0,3	0,1	−0,7	1,5	−1	−1	−0,1	−0,3	0,6	−0,3	−0,6	1,3	0,7	−0,6	−1,8	**11**
C4 LOW	1,2	1,6	1,2	0,9	0,7	0,7	1,8	1,2	1,3	2,3	0,5	1,8	2,1	0,7	−2,3	−0,2	−1,1	−0,4	−0,5	2,3	**6,7**
C4 HIGH	0,4	1,9	1,2	1,3	1,9	3,3	2,3	1,0	2,3	1,4	1,3	1,9	3,2	−1,2	−1,4	0,2	0,1	−1	1,0	0,4	**6,7**
C3 LOW	−3,2	−0,4	−0,9	−0,9	−0,9	0,5	−1,4	−0,2	−1,3	−0,8	−4,5	−2,7	0,0	−5,4	−5,4	**−7,2**	−5,4	−5	−4,5	−4,0	**5,5**
C3 HIGH	−2,4	−0,7	−0,8	−0,8	−1	1,09	−1,4	−1,8	−1,4	−0,6	−2,2	−1,4	0,27	−2,6	−5,2	−2,7	−4,1	−3,2	−2,0	−3,1	**5,5**

LCT, límite de cambio total; “-“ no se dispone de resultados ya que, para la ApoA, no se estudió el nivel 2 del LL por no presentar una concentración adecuada y no ser utilizado como control interno de esta magnitud.

**Figura 3:**
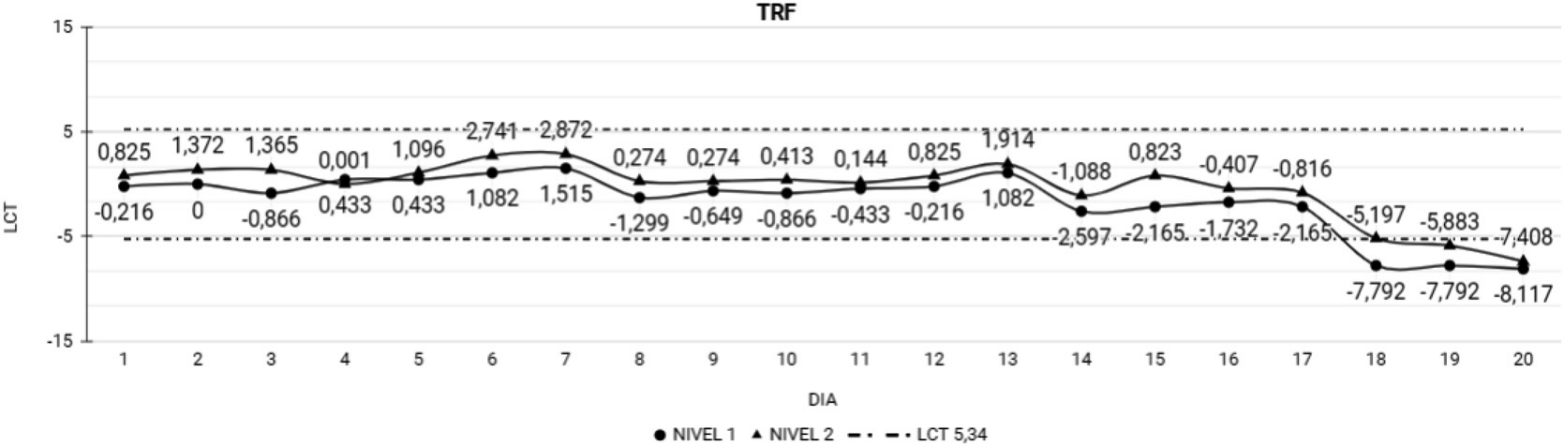
Magnitudes que perdieron la estabilidad en el CC LI. Magnitudes que perdieron la estabilidad en el CC LI durante los 20 días del estudio. TRF, transferrina; LCT, límite de cambio total; Nivel 1, nivel bajo del CC LI; Nivel 2, nivel alto del CC LI.

Por otro lado, con respecto a los CC LI, LL 8 de las 9 magnitudes fueron estables durante los 20 días del estudio. La Trf perdió la estabilidad el día 18 con una tendencia descendente (Figura 3).

En el caso de la magnitud ALT analizada en el MQ y el C3 analizado en el LI, tan sólo un dato puntual de Xt % excedió el LCT en un solo nivel de CC por lo que no se consideró significativo.

## Discusión

En el presente estudio hemos podido verificar el tiempo de estabilidad para cada magnitud bioquímica analizada en los CC almacenados en el MR de AS. Las 48 magnitudes estudiadas fueron estables durante un mínimo de 7 días según el criterio de estabilidad LCT. Este criterio ha sido utilizado en estudios anteriores donde se analizó la estabilidad, en la fase post-analítica, de muestras de suero de pacientes almacenadas en neveras automatizadas y conectadas a una cadena de automatización [3, 4]. Sin embargo, en el presente estudio se han analizado muestras de control interno que son almacenadas en el MR del analizador, influyendo directamente en la fase analítica.

Según nuestro conocimiento tan solo existen dos analizadores de bioquímica en el mercado que incorporen un sistema de refrigeración automatizado permitiendo el almacenamiento de CC en su interior y la programación automática de los mismos. Estos sistemas son AS de Siemens y Alinity de Abbott (Alemania). La diferencia entre ambos es que AS tiene un MR exclusivo para almacenar CC y calibradores refrigerados y tapados, mientras que en Alinity los CC y calibradores se guardan en el compartimento de reactivos y no se guardan tapados.

El módulo MR permite ahorrar tiempo de dedicación del personal técnico y facilita el trabajo dentro del laboratorio pudiendo ser programados los CC a las horas de menos volumen de muestras. Sin embargo, conocer la estabilidad de dichos CC almacenados en el MR es fundamental para asegurar la calidad analítica. Hasta la fecha y según nuestra búsqueda bibliográfica, no existe ningún trabajo previo publicado sobre la estabilidad de los controles internos almacenados en el MR de AS.

En el presente trabajo no se utilizaron los materiales control InteliQ de Bio-Rad porque, en el momento del estudio, no se disponía aún de ellos. No obstante, creemos que los resultados obtenidos son aplicables a los actuales controles InteliQ ya que el material de control es el mismo, sólo cambia el contenedor pasando de vial a tubo de 5 mL que es el contenedor utilizado en el estudio. Por otra parte, en las instrucciones de uso (IFU) de los CC utilizados se indica el tiempo de estabilidad que coincide con el que se indica en el IFU de los CC InteliQ. Según Bio-Rad en los CC MQ todos los analitos son estables 14 días, menos ALP, AST y TBil que son estables 9 días y DBIL, cHDL, CK, IP y Trig que son estables 7 días. Sin embargo, los resultados del presente estudio demostraron que todas las magnitudes fueron estables durante al menos 15 días excepto la ALP que perdió la estabilidad a partir del día 8, AST el día 10 y Ca, LDH y TBil el día 13. En cuanto al CC UC, Bio-Rad indica en su IFU que todas las magnitudes son estables durante 30 días, no obstante en el estudio se analizó el CC UC durante 15 días observando que CL y Glu perdían la estabilidad a partir del día 8 según el criterio LCT. Por último, en los CC LI y LL según el IFU de Bio-Rad todas las magnitudes del estudio son estables durante 30 y 14 días respectivamente. Sin embargo, los resultados del estudio demostraron que ambos CC fueron estables durante al menos 20 días a excepción de la Trf del LI que perdió la estabilidad el día 18. Estos resultados, demuestran la necesidad de comprobar la estabilidad de los CC según los criterios establecidos por cada laboratorio.

Tras los resultados obtenidos podemos concluir que el sistema de refrigeración MR de AS conserva de forma adecuada los CC de bioquímica que se han estudiado. Además, conocer el tiempo de estabilidad para cada magnitud permite personalizar el tiempo máximo de utilización del CC por magnitud y no por material de control. Finalmente, este conocimiento posibilita la correcta valoración de los CC por parte del personal del laboratorio, minimizando el número de repeticiones o cambio de material control ante resultados desviados.

### Limitaciones

El estudio presenta una limitación analítica debida al posible sesgo como consecuencia de que los materiales control utilizados durante el estudio fueron analizados en distintas series analíticas. Según el artículo publicado por Fernández-Calle P. et al. [7] se tendrían que haber realizado alícuotas, congelarlas y posteriormente analizar las muestras en una misma serie analítica. Sin embargo, estas recomendaciones se publicaron en 2019 que es una fecha posterior a la realización del estudio.
